# Mining Genetically Encoded Biosensors from Filamentous Fungi

**DOI:** 10.3390/jof12020150

**Published:** 2026-02-19

**Authors:** Shuhui Guo, Shaozheng Song, Zhunzhun Liu, Yunjun Ge, Ye Chen

**Affiliations:** 1 School of Medicine & Health Sciences, Wuxi Taihu University, Wuxi 214064, China; ssz0610@163.com (S.S.); liuzz@wxu.edu.cn (Z.L.); 2 State Key Laboratory of Quantitative Synthetic Biology, Shenzhen Institute of Synthetic Biology, Shenzhen Institutes of Advanced Technology, Chinese Academy of Sciences, Shenzhen 518055, China; 3 Wuxi School of Medicine, Jiangnan University, Wuxi 214122, China; geyunjun@jiangnan.edu.cn

**Keywords:** genetically encoded biosensor, filamentous fungi, synthetic biology, transcription factor-based biosensor, GPCR, photoreceptor, sensing element

## Abstract

Genetically encoded biosensors represent cutting-edge biosensors due to their capabilities in real-time monitoring and precise control in living cells. However, the development of eukaryotic genetically encoded biosensors for new analytes is constrained by the shortage of signal–receptor pairs. Bacterial biosensors have been transferred to eukaryotes to expand the signal detection space, which has achieved remarkable success. However, due to the significant differences between eukaryotic and prokaryotic gene expression systems, optimizing bacterial biosensors has proven challenging. Successful cases indicate that developing orthogonal signal–receptor pairs directly from eukaryotic systems may offer a viable solution. Indeed, the potential of filamentous fungi—a highly diverse group of organisms that share conserved as well as specific signaling and metabolic pathways with yeast and mammalian cells—has been largely overlooked in biosensor development. In this review, we systematically examine biosensing systems in filamentous fungi, summarize their signal recognition receptors, signal transduction pathways, responsive transcription factors, and provide an overview of the biosensors and synthetic tools developed from them. Finally, we highlight the promise and challenges of biosensor development from filamentous fungi and discuss their potential applications.

## 1. Introduction

Genetically encoded biosensors are a class of biomolecular devices capable of transducing biological recognition events into detectable signals such as fluorescence or gene expression [1,2]. Traditional physical/chemical probes (measuring metabolites, temperature, pH, and dissolved oxygen) cannot directly convert detected signals into downstream biological events and are thus limited to monitoring and cannot achieve automatic regulation. As integral components of living cells, genetically encoded biosensors can enable not only real-time and noninvasive monitoring of intracellular physiological and biochemical states but also precisely control and program higher-order biological processes to confer cellular intelligence [1,3,4,5,6]. Moreover, through subcellular localization design, high spatial resolution within cells can be achieved [7]. Genetically encoded biosensors can be stably inherited, ensuring sustainability at low costs once successfully designed and constructed [8]. Ideally, a versatile biosensor should display a large dynamic range (ON/OFF ratio), minimal leakiness, high inducibility, high host orthogonality (i.e., it does not interfere with host endogenous systems and vice versa), sustainable expression, rapid inducer response, and nontoxicity to the host [9].

**Figure 1:**
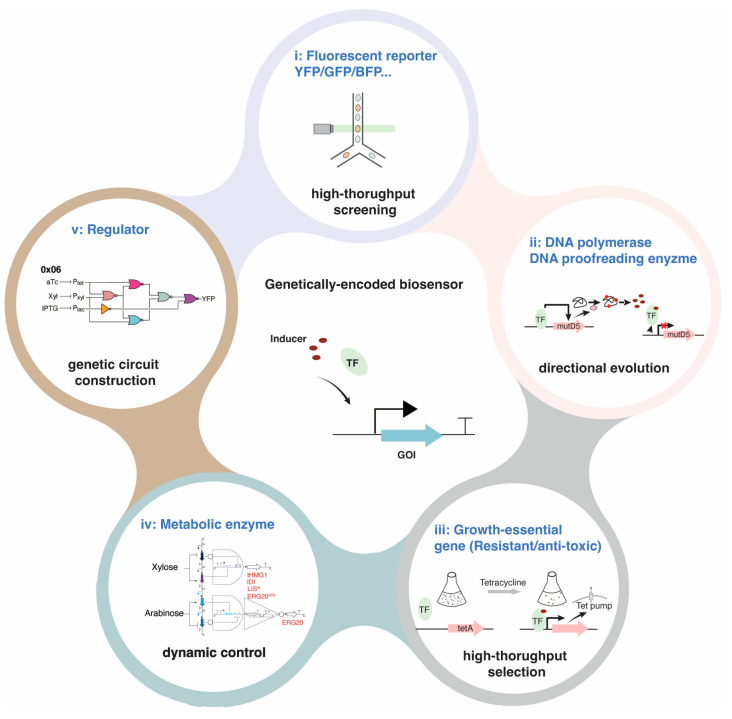
Diverse output genes and their corresponding applications by a TF-based biosensor.

Generally, a genetically encoded biosensor primarily comprises a signal recognition module, a transduction module and a signal output module [10]. Signal recognition elements are critical for the development of biosensors. In prokaryotes, signal recognition is typically mediated by allosteric transcription factors (TFs) [11], aptamers [12,13] and Two-Component Systems (TCSs) [14]. In fungi, signal recognition elements include membrane-bound G protein-coupled receptors (GPCRs), ion channels, TCSs and zinc cluster TFs [15,16,17]. Currently, genetically encoded biosensors are mainly constructed based on TFs, as TF-based biosensors offer the versatility to regulate diverse downstream target genes (Figure 1). Indeed, TF-based biosensors serve as core components in synthetic biology, with broad applications in high-throughput screening and selection, directed evolution, dynamic regulation of metabolic pathways, and construction of synthetic genetic circuits [1,18,19]. Recently, synthetic design and construction based on filamentous fungal GPCRs and photoreceptors have also been reported [20,21,22].

With the increasing complexity and refinement of biomanufacturing processes, coupled with the growing demand for point-of-care diagnostics in biomedical applications, there is an urgent need for more effective process control strategies and an expanded detectable signal space. The rapid development of biosensors offers powerful tools for advancing the frontiers in these areas.

## 2. Limitations of Existing Yeast Biosensors for Synthetic Design

The commonly used yeast biosensors can be classified into endogenous induction systems and heterologous bacterial-derived biosensors. Each category has inherent limitations and developmental bottlenecks for synthetic design.

**Figure 2:**
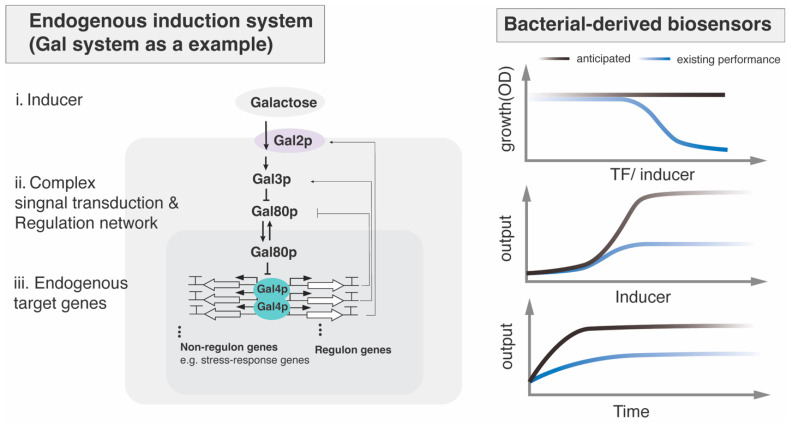
Limitations of endogenous and bacterial-derived biosensors.

The intensively used induction systems in metabolic engineering are dominated by endogenous yeast biosensors, including the galactose-responsive GAL system, the copper-inducible P_CUP1_ system, the phosphate-responsive P_PHO5_ system and the methionine-regulated P_MET3_ system [9]. The advantages of these systems are evident, including ease of molecular operation and high inducibility. However, the portability and application scenarios of these native systems are constrained by complex signal transduction cascades, their coupling with host growth requirements and off-target effects (non-orthogonality) [23,24,25] (Figure 2). In the past decades, to overcome the limitations of endogenous biosensors, bacterial-derived biosensors have been transferred to yeast and optimized through directed evolution [26,27]. Engineering prokaryotic regulators is an effective strategy to enrich the yeast biosensor toolbox. However, progress is constrained by the limited number of identified bacterial biosensors and host compatibility issues such as growth toxicity, low inducibility and slow induction speed [20,28,29]. Although fusing strong Activation Domains (ADs) to bacterial TFs may offer a solution, these synthetic systems often exhibit high leakiness, especially when multiple binding sites are present in tandem in the promoter [30]. No rational strategy has been developed beyond directed evolution to optimize bacterial transcriptional activator-based biosensors, as the activation mechanism is different from that of eukaryotes.

In summary, non-orthogonal endogenous induction systems, underperforming bacterial-derived biosensors and the shortage of characterized signal-receptor pairs have hindered the development of genetically encoded biosensors for synthetic design.

## 3. Emerging Opportunity: Mining Biosensor from Filamentous Fungi

Fungi serve as important eukaryotic models in eukaryotes for studying environmental perception and signal transduction. As ecological decomposers, filamentous fungi exhibit remarkable adaptability and the ability to sense a wide array of signals, including pheromones, nutrients, gases, light, host organisms, and mechanical stress [15].

Filamentous fungi have evolved to possess a rich repertoire of signal receptors, including GPCRs, zinc cluster TFs, photoreceptors and TCSs. First, GPCRs mediate the detection of nutrients, pheromones, and various types of stress. There are over 15,000 GPCRs predicted in Ascomycota, while only three GPCRs—Ste2, Ste3 and Gpr1—have been characterized in yeast [31]. In other words, species in Ascomycota have an average of over 30 GPCRs. Second, it has been reported that fungi lack nuclear receptors, while zinc cluster TFs may represent their functional analogs [17]. Filamentous fungi possess a unique capacity to utilize some nutrients, like pentoses, and biosynthesize secondary metabolites when compared to yeast. Like nuclear receptors, increasing evidence shows that fungal zinc cluster TFs are direct small-molecule sensors [32,33,34,35]. For example, in ascomycetous fungi, a network of regulators has been reported to control biomass degradation and catabolic pathways for the resulting monomers, which are often the inducers of these regulators’ activity [32,36]. Moreover, filamentous fungi are renowned for drug discovery due to the rich genetic resource they provide for secondary metabolite biosynthesis [37]. Their pathway genes are clustered and often include a specialized transcriptional activator that drives high-level expression of key metabolic enzymes [38]. Most of these metabolic TFs belong to the Zn(II)_2_Cys_6_ family, and their DNA-binding domains (DBDs) exhibit remarkable sequence recognition specificity. This specificity is dictated by the following molecular mechanisms: small-molecule-induced activation (e.g., proline-induced DNA binding of PrnA [39]) and signal-induced homo- or heterodimerization [34]. Moreover, *Saccharomyces cerevisiae* lacks native metabolic pathways and regulatory networks for utilizing certain carbon sources like pentoses (e.g., xylose, arabinose and rhamnose) or synthesizing secondary metabolites, which indicates that heterologous reconstruction of these pathway-specific TF-mediated induction systems could be suitable for synthetic design. Third, unlike blind yeast, filamentous fungi, especially Ascomycota species, are capable of perceiving a broad spectrum of light. Taking *Botrytis cinerea* as an example, there are at least 11 potential photoreceptors capable of perceiving near-UV, blue, green, red and far-red light [40]. Filamentous fungi hence provide prolific genetic components for optogenetic tools. Lastly, filamentous fungi have been reported to possess far more histidine kinases (HKs) than yeasts. Only one HK, Sln1, has been identified in *S. cerevisiae*, while at least 10 HK genes are predicted in filamentous fungi such as *Neurospora crassa*, *Cochliobolus heterostrophus*, *Gibberella moniliformis* and *Botryotinia fuckeliana* [41].

As mentioned above, compared to yeast, filamentous fungi possess unique capabilities in broader nutrient degradation, secondary metabolite synthesis and light sensing. It is reasonable to assume that the sensing receptors and regulators involved in these processes are unique to filamentous fungi and hence may exhibit orthogonality when introduced into yeast. On one hand, this orthogonality ensures that yeast growth is not significantly impaired by the introduced biosensor. On the other hand, the output behavior of the introduced biosensor would be predictable, as it would not be affected by the yeast endogenous system. Indeed, this hypothesis has been preliminarily validated [20]. Beyond orthogonality, mining biosensors from filamentous fungi offers the following advantages. First, many zinc cluster TFs act as transcriptional activators. Activator-based biosensors typically achieve higher induction levels and larger dynamic ranges than those based on bacterial repressors [20]. Furthermore, they exhibit a more predictable positive correlation with TF abundance [42]. Second, molecular genetics between filamentous fungi and budding yeast are conserved [43,44], including gene expression machinery, transporters, signaling transduction and primary metabolic pathways. In other words, when transferring a response system from filamentous fungi to *S. cerevisiae*, the signal-specific components are sufficient to confer functionality, as evidenced by the xylose and arabinose sensors XlnR and AraR [20]. Third, there is ease of molecular manipulation. In our experience, codon optimization is not required for heterologous expression of filamentous fungal-derived genes in *S. cerevisiae*. However, this conservation in signaling pathways would be a double-edged sword, as it may also interfere with the endogenous systems of yeast. Therefore, further work is needed to first determine whether interference exists. If interference is present, it should be quantified to define the potential scope of application.

## 4. Native Biosensors in Filamentous Fungi

As key decomposers, filamentous fungi possess the ability to detect and degrade complex plant biomass, whereby they sense breakdown products (monomeric nutrients) and activate intricate signaling cascades to induce downstream gene expression for further catabolism. Moreover, to adapt to fluctuations in pH, temperature, light, moisture, and oxygen availability, filamentous fungi have evolved specialized sensors—including transmembrane receptors, TFs and phytochromes—that detect these environmental cues and initiate adaptive survival strategies. Consequently, filamentous fungi naturally harbor a rich repertoire of small-molecule and environmental-factor biosensors. A thorough understanding of these signaling mechanisms provides the foundation for cross-species transfer and the rational engineering of these native components into synthetic biological devices.

### 4.1. Small-Molecule Induction Systems

In fungi, small-molecule sensors function either as membrane receptors or directly as zinc cluster TFs. Examples in *S. cerevisiae* include glucose sensors (Snf3, Rgt2 [45] and Gpr1 [46]), galactose sensor Gal3 [47], phosphatidate sensor Opi1 [48], amino acid sensors (Ssy1 [49] and Gap1 [50]), cysteine sensor Pib2 [51], and α-isopropylmalate (α-IPM) sensor Leu3 [52]. In filamentous fungi, responsive TFs and GPCRs for small molecules are summarized in Table 1 and Table 2. In the sections below, we focus on the cAMP-PKA signaling pathway and the reported TFs that respond to specific small molecules.

#### 4.1.1. Small-Molecule Signaling Pathways

**Figure 3:**
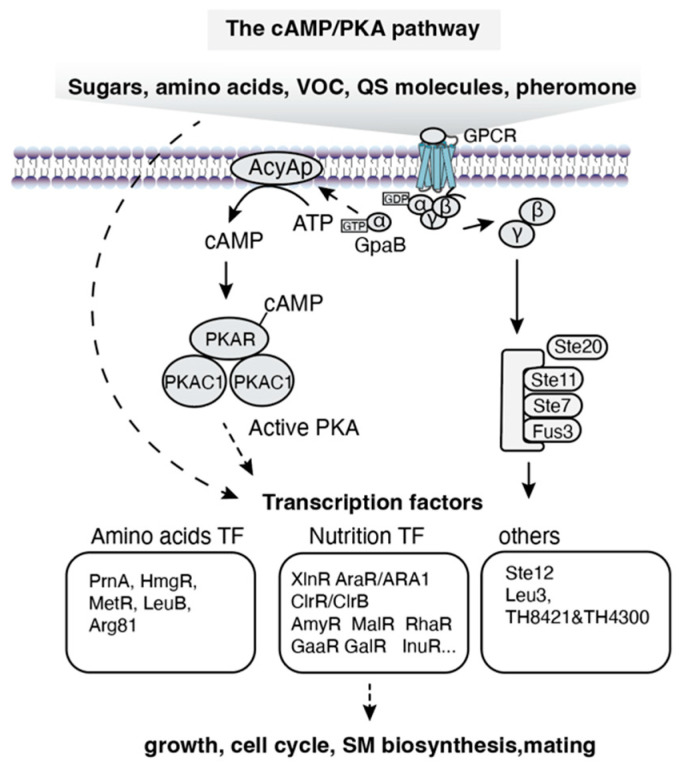
Small-molecule signaling pathway in filamentous fungi.

Small molecules sensed by filamentous fungi include carbon sources, amino acids, volatile organic compounds (VOCs), quorum-sensing molecules and pheromones. The specific signal transduction pathways and the resulting biological functions vary depending on the type of signal molecules and organisms, though their core upstream components share evolutionary conservation with yeast—most notably the cAMP-PKA pathway (Figure 3) [53]. When extracellular small molecules exceed a concentration threshold, membrane-localized GPCRs detect these ligands, triggering dissociation of the Gα subunit (e.g., Aspergillus GpaB [54]) or Gβγ. The free Gα-GTP then diffuses to adenylate cyclase (ACYA [55]), catalyzing production of cAMP and subsequent activation of protein kinase A (PKA). On the other hand, pheromone peptide-triggered Gβγ dimer release initiates the MAPK cascade. These complex regulatory networks ultimately converge on the phosphorylation of specific effector TFs (Figure 3). Presumably, the conservation of cAMP-PKA signaling could provide a foundation for simplified cross-species biosensor transfer across fungal species.

#### 4.1.2. Small-Molecule-Responsive TFs

Many small-molecule-responsive TFs have been identified in filamentous fungi (Table 1). Most of them are involved in nutrient utilization [36] and community communication, for example, different types of carbon sources, including monomers of plant biomass degradation and amino acids; fungal species-specific GPCR-mediated pheromones for sexual reproduction; volatile compounds and small molecules for inter- or intra-species communication (i.e., quorum sensing). Based on these identified sequences of TF proteins and DNA-binding motifs, they are expected to be applied in biosensor development in other eukaryotic species.

**Table 1:** Small-molecule-responsive TF summary.

Inducer	Responsive TF	UAS	Species	Biosensor Developed?	Ref.
Plant biomass degradation monomer					
xylose	XlnR	GGCTAAA	*Aspergillus* spp., *Penicillium* spp.	yes	[20,56]
arabinose	AraR	N/A *	*Aspergillus* spp.	yes	[20,57]
maltose	MalR	N/A	*A. oryzae*	no	[58]
sucrose/inulin	InuR	CGG-X_8_-CGG	*A. niger*	no	[59]
rhamnose	RhaR	CGG-X_11_-CCG	*A. nidulans*	no	[60]
cellobiose	ClbR	CGG or CCG	*A. aculeatus*	no	[61]
D-galacturonic acid	GaaR	TCC-X_1_-CCAAT	*A. niger*	no	[62]
isomaltose	AmyR	CGG-X_8_-CGG or CGGAAATTTAA	*A. nidulans*	no	[63]
L-fucose	FUR1	CCGACGG	*T. reesei*	no	[64]
β-mannan	ManR	CAGAAT	*A. oryzae*	no	[58]
ferulic acid	FarA	CCTCGG	*A. niger*	no	[65]
D-Fructose	FruR	TGAWWGWTTT	*F. prausnitzii*	no	[66]
L-acetic acid	Haa1	SMGGSG	*S. cerevisiae*	no	[67]
NO^3−^ or NO^2−^	NirA	CTCCGHGG	*Aspergillus* spp.	no	[68]
		Amino Acids			
proline	PrnA	CCGG-N-CCGG	*A. nidulans*	no	[39]
tyrosine	HmgR	N/A	*A. fumigatus*	no	[69]
valine/leucine/isoleucine	LeuB	CCG-X_4_-CGG	*Aspergillus* spp.	no	[70]
arginine	ARCA	CTGCACTTAGAG	*A. nidulans*	no	[71]
methionine	MetR	ATGRYRYCAT	*A. nidulans*	no	[72]
Pheromone					
pheromone	Ste12, SteA	TGAAACA	*Aspergillus* spp., *C*. *albicans*	yes	[73]
Volatile Compounds (VOC)					
aromatic compounds (o-toluidine, guaiacol)	TH8421&TH4300	TH8421: CGG-X_10_-CCG, CGG-X_5_-CGG; TH4300: GG-X_6_-CGG	*T. hirsuta*	no	[34]
acetaldehyde	AlcR	half site: TGCGG	*A. nidulans*	yes	[74,75]
Quorum Sensing Molecule (QSM)					
Farnesoic acid	Hot1	TTAATAATCAAAAACAATTTAATCGT	*C. albicans*	no	[76]
Farnesol	Czf1/Efg1(APSES TF)	CZF1: TTWRSCGCCG; Efg1: TGCAT	*Candida* spp.	no	[77]
Isoamyl alcohol	Aro80	CCG-X_7_-CCG	*C. albicans*	no	[78]
1-dodecanol	Sfl1	AGAA-X-TTCT	*C. albicans*	no	[79]
QSP1 (QS peptide)	Cqs2	N/A	*C. neoformans*	no	[80]
Other					
Quinic acid	QF-QS	GGRTAARYRYTTATCC	*N. crassa*	yes	[81,82]

N/A *: Not available.

### 4.2. Environmental Cue-Responsive Systems

Environmental signals are initially perceived by receptor proteins—such as GPCRs, ion channels or HKs—located on the cell membrane. These signals are then transduced through distinct signaling pathways like MAPK pathways and amplified via signaling cascades, and ultimately lead to alterations in the activity of TFs, thereby driving physiological adaptations to environmental perturbations. Accordingly, the following sections will discuss key components—including GPCRs, signal transduction pathways, and TFs—in the context of different environmental stimuli.

#### 4.2.1. GPCRs Responsive to Environmental Cues

GPCRs represent the largest class of cell surface receptors in eukaryotes, and GPCR signaling serves as a primary mechanism for eukaryotic cells to perceive and respond to environmental cues. These receptors are involved in sensing nutrients, hormones, light, pH, temperature, and inter-population signaling. Environmental cue-related GPCRs, along with their potential ligands, physiological functions, and species distribution, are summarized in Table 2.

**Table 2:** Environmental cue-responsive GPCR summary.

GPCR	Signal	Physiological Role/Evidence	Species	Ligand Validated GPCR	Ref.
Nutrition					
Gpr1, Git3, GPR-4, GprC, GprD	carbon source	sense the carbon source and further affect growth and metabolism	*S. cerevisiae*, *S. pombe*, *N. crassa*, *Aspergillus* spp.	ScGpr1	[45,83,84,85]
CnGpr4	methionine	upstream of the cAMP–PKA pathway and regulates methionine-induced mating and contributes to capsule formation	*C. neoformans*	CnGpr4	[86]
GprK, GPR-7 (NCU09883)	pentose	Δ*gprK* mutant is restricted on the medium when pentose is the sole carbon source	*A. fumigatus*, *N. crassa*		[87,88]
GprH	tryptophan, glucose	the absence of GprH results in a reduction in cAMP levels and PKA activity upon adding glucose or tryptophan to starved cells	*A. nidulans*		[89]
Stm1, GPR-5 (NCU00300), GPR-6 (NCU09195), GprR	nitrogen source (e.g., arginine, ornithine)	expression was induced by N starvation signal through Gpa2(G_α_)	*S. pombe*, *N. crassa*, *A. flavus*		[88,90,91]
Hormone					
Ste2, GprA, PRE-1 (NCU00138), Cpr2, Ste3a	α-factor pheromone	mediate cell cycle arrest and cell fusion with the opposite mating type	*S. cerevisiae*, *A. nidulans*, *N. crassa*, *C. neoformans*	ScSte2, CaSte2	[92,93,94,95,96]
Ste3, GprB, PRE-2(NCU05758), Ste3α	a-factor pheromone	mediate cell cycle arrest and cell fusion with the opposite mating type	*S. cerevisiae*, *A. nidulans*, *N. crassa*, *C. neoformans*	ScSte3, CaSte3	[93,97,98,99]
Gpr-12	abscisic acid (ABA)	could be involved in signaling related to abscisic acid	*N. crassa*		[88,100]
Light					
NOP-1, CarO	green light (534&561 nm)	Light-controlled sexual development	*N. crassa*, *F. fujikuroi*	NOP-1, CarO	[101,102]
ORP-1 (NCU01735)	N/A	N/A	*N. crassa*		[101,103]
Oxidative stress					
GprH	N/A	loss of *gprH* rendered the fungus more resistant to H_2_O_2_	*A. flavus*		[91]
pH					
PalH/Rim21, GprM, GprR	alkalne pH	palH GPCR-arrestin (Rim8) signaling; null mutant of GprM and GprR result in remarkable morphogenetic alteration	*A. flavus*		[91,104]
GprD, GprF, GprG, GprK, GprM	acid pH	null mutant of these GPCRs was more resistant to acidic pH	*A. flavus*		[91]
Osmotic stress					
GprK, GprM, GprR		null mutants of GprK, GprM, and GprR were more sensitive than the wild-type to hyperosmotic conditions	*A. flavus*		[91]
Temperature					
GprC, GprD, GprF, GprG, GprO	thermal stress	expression of these GPCRs was significantly different at three temperatures (20 °C, 28 °C, and 37 °C); growth defects in null mutants of GprC or GprD were found to be temperature dependent	*A. flavus*, *At. fumigatus*		[105,106]
Inter-communication signal					
Gpr1, Gpr2, Gpr3, GprC	ascaroside	trigger nematode trapping	*A*. *oligospora*, *A. flagrans*	AoGprC, AfGprC	[107,108]
CaGpr1	l-lactic acid	involved in Lactate signaling and regulates fungal β-glucan masking and immune evasion	*C. albicans*		[109]
GprC, GprD	linoleic acid derivates (e.g., 9-HODE, 13-HODE)	quorum-sensing receptors	*Aspergillus* spp.		[91,110]
GprO, GprP	13(S)-HpODE	oxylipin sensing	*A. flavus*		[91]
Pth11, MrGpr8 (Class X); GPR-15~39	hydrophobic surfaces	the CFEM domain is essential for sensing hydrophobic surfaces and fungal virulence against plants and insects	*Magnaporthe* spp., *N. crassa*		[88,111,112,113]

#### 4.2.2. Signaling Pathway of Environmental Cues

**Figure 4:**
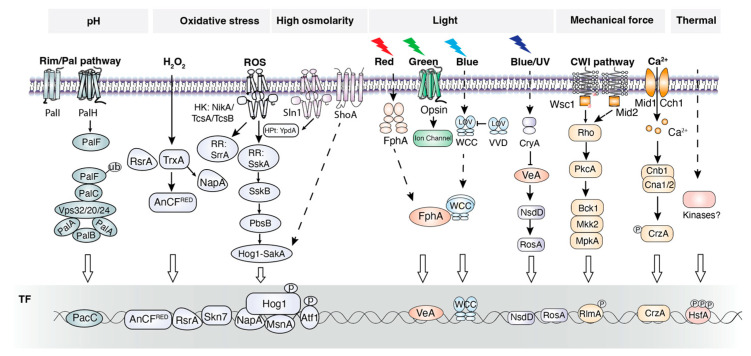
Signaling pathway for environmental cues in filamentous fungi.

Filamentous fungi have evolved diverse survival strategies and signaling pathways to sense these perturbations and adapt to fluctuating environments (Figure 4) [15]. The core signaling pathways responding to environmental cues in filamentous fungi include the TOR-MAPK, HOG-MAPK, cell wall integrity (CWI)-MAPK, Ca^2+^ signaling pathway, and HK-MAPK pathways [43]. These MAPK pathways are conserved between filamentous fungi and yeasts [44]. MAPK pathways are interconnected with other signaling pathways, particularly the cAMP-PKA one [114]. Given the complexity of signal networks involved in environmental sensing, this review focuses on well-characterized signaling pathway components in filamentous fungi.

The Rim/Pal pathway is a fungal-specific and well-studied signaling pathway that responds to extracellular pH changes [115]. This pathway is conserved in filamentous fungi, at least within the Ascomycota phylum, and shares similarities with *S. cerevisiae*. As the Rim101 ortholog in yeast, PacC in the Aspergillus species serves as the central TF mediating pH signaling and requires two-step proteolytic processing to become functional [116].

Environmental cues such as oxygen levels, mechanical damage, and different nutrient types and abundances trigger intracellular oxidative stress responses. Current research indicates that oxidative stress signaling in *S. cerevisiae* and Aspergillus species is highly conserved and involves both the canonical HK-Hog1-AtfA (AP-1 ortholog) pathway and non-canonical pathways mediated by SrrA (Skn7 ortholog) and NapA (Yap1 ortholog) [117]. These pathways differ in their reactive oxygen species (ROS, e.g., H_2_O_2_, O^•−^) sensors and downstream TFs. Moreover, these TFs are functional across species of yeast and filamentous fungi [118].

Unlike *S. cerevisiae*, which lacks light-sensing capability, filamentous fungi are generally capable of perceiving light and can distinguish different wavelengths. Several photoreceptors for specific light spectra have been characterized (Table 3). These include cryptochrome CryA for near-UV light, WC-1 and Vivid for blue light, opsin for green light and phytochrome FphA for red light [119]. These photoreceptors exhibit distinct subcellular localizations (e.g., opsin on the plasma membrane, WC-1 in the nucleus, and FphA in the cytoplasm). Except for WCC complexes (WC-1 and WC-2), the downstream signal transduction mechanisms of these photoreceptors remain poorly understood. Triggered by blue light, the flavin-cysteinyl adduct is formed in the LOV domain to expose AD and stabilize the WC-1/WC-2 dimer, thereby translocating to the nucleus and functioning as a TF. The Hog1-Atf1 pathway has been reported to participate in FphA-mediated red light signaling [119]. The signaling mechanisms of cryptochromes (e.g., CryA) in response to blue light remain largely unexplored in fungi. In *Arabidopsis thaliana*, cryptochrome CryA directly binds the TF—CIB1 upon blue light stimulation to activate transcription initiation [120]. This example may provide insights into CryA signaling mechanisms in fungi. Green light receptors like NOP-1 have been identified in filamentous fungi. Green light induces photoisomerization of retinal from all-trans to 13-cis, which activates coupled pump activity. The guanylyl cyclase effector domain in rhodopsin may couple light sensing with the production of the second messenger cGMP [121].

Microorganisms inherently exist in mechanically dynamic environments, where CWI is crucial for maintaining osmotic homeostasis and resisting external mechanical forces. Membrane proteins Wsc1 and Mid2 function as mechanosensors; their extracellular domains undergo spring-like extension and compression, thereby initiating intracellular signaling. Additionally, the calcium channel proteins Mid1 and Cch1 are capable of perceiving mechanical stress and membrane damage. Downstream signaling pathways—including the CrzA-Ca^2+^ pathway, the CWI-MAPK cascade, and the SmuSH pathway—act in coordination to mediate responses to sustained mechanical pressure [122]. By contrast, research on fungal sensors for temperature stimuli—both heat and the cold—remains notably limited.

**Table 3:** Photoreceptors in fungi.

Photoreceptor	Protein Domain	Chromophore	Species	Developed Photoreceptor	Ref.
LOV					
WC-1, WC-2	LOV, PAS, GATA DBD, AD	FMN, FAD	*N. crassa*	WC-1	[123]
BcWCL1, BcWCL2	LOV, PAS, GATA DBD, AD	FMN, FAD	*B. cinerea*		[124]
FaWC1, FaWC2	FaWC1: LOV, PAS, NLS, ZnF, Poly-Q FaWC2: PAS, NLS, ZnF	FAD(FaWC1)	*F. asiaticum*		[125]
PoWC-1, PoWC-2	PoWC1: PAS, ZnF, LOV PoWC2: PAS, GATA-ZnF	FAD(PoWC-1)	*P. ostreatus*		[126]
SfWC-1	LOV, PAS, GATA-ZnF, Poly-Q, NLS	FAD	*S. fimicola*		[127]
BLR-1, BLR-2	BLR-1: LOV, PAS, GATA-ZnF, NLS, Poly-Q PoWC2: PAS, GATA-ZnF	FMN	*T. atroviride*		[128]
LreA, LreB	LreA: LOV, PAS, GATA-ZnF, NLS LreB: PAS, GATA-ZnF	FAD (LreA)	*A. nidulans*		[129]
LreA	LOV, PAS, GATA-ZnF, NLS	FAD, FMN	*A. alternata*		[130]
MadA, MadB	MadA: LOV, PAS, GATA-ZnF, MAPK MadB: PAS, GATA-ZnF, WRKY	FAD (MadA)	*P. blakesleeanus*		[131]
Vivid	N/C-cap, LOV	FAD, FMN	*N. crassa*, *B. cinerea*, *T. reesei*	*N. crassa* Vivid	[132,133,134]
BcLOV3, BcLOV4	BCLOV3: short-LOV BCLOV4: RGS, LOV	FAD, FMN	*B. cinerea*	BcLOV4	[135]
Opsin/Rhodopsin					
NOP-1	Seven transmembrane, helix retinal-binding protein, low pump activity	Retinal (11-cis-Retinal)	*N. crassa*		[136]
CarO	Seven transmembrane, Green light-driven proton pump	all-trans-retinal	*F. fujikuroi*		[102]
Opsin-1, Opsin-2	Green light-driven proton pumps		*U. maydis*		[137]
Opsin	Seven transmembrane, pump activity	9- cis-retinal isomer	*S. punctatus*		[138]
BcOPs (BOP1, BOP2)	Seven transmembrane	all-trans-retinal	*B. cinerea*		[40]
Opsin-1, Opsin-2			*B. oryzae*		[139]
PhaeoRD1, PhaeoRD2	Seven transmembrane; pump activity		*P. nodorum*		[140]
ApOps1, ApOps2, ApOps3			*A. pullulans*		[141]
OpsA	no pump activity		*F. fujikuroi*		[142]
Rhodopsin-guanylyl cyclases (RhoGCs)					
BeGC1	Rhodopsin domain, Guanylyl cyclase domain	retinal	*B. emersonii*		[143]
RGC1, RGC2, RGC3(NeoR)			*R. globosum*		[144]
Cryptochromes					
Neurospora CRY	photosensing; FAD-binding domain; PHR; CCE	FAD, MTHF	*N. crassa*		[145]
Cry1	N-terminal DNA photolyase, FAD-binding domain, C-terminal extension domain	FAD	*T. atroviride*		[146]
Cry1		FAD	*T. reesei*		[147]
CryA	The PHR domain, FAD-binding domain, C-terminal extension domain	FAD	*A. nidulans*		[148]
CryD	FAD binding domain, photolyase domain	FAD, MTHF	*F. fujikuroi*		[149]
BcCRY1, BcCRY2		FAD	*B. cinerea*		[150]
CryA		FAD, MTHF	*P. blakesleeanus*		[151]
Phytochromes					
XccBphP	PAS2, GAF, PHY, PAS9	bilin, biliverdin, phycoerythrobilin, phytochromobilin	*X. campestris*		[152]
MmBphP	PAS, GAF, PHY		*M. magneticum*		[153]
Agp1, Agp2	Agp1: PAS, GAF, PHY, HisK, ATPase Agp2: PAS, GAF, PHY, HWE-HK		*A. fabrum*		[154]
FphA	P2, GAF, PHY, HKD, RRD		*A. nidulans* *A. alternata*	AnFphA	[155,156]
Phy1	PAS, GAF, PHY, HKD, RRD		*U. maydis*		[157]
PHY-1, PHY-2	PLD, GAF, PHY, HKD, RRD		*N. crassa*		[158]
BcPHY-1, BcPHY-2, BcPHY-3	PAS, GAF, PHY, HK, ATP, RRD		*B. cinerea*		[40]

#### 4.2.3. TFs Responsive to Environmental Cues

A set of TFs responsive to specific environmental cues has been identified (Table 4). How do these TFs perceive their cognate signals? What are their regulons or DNA-binding motifs? Most importantly, do they hold potential for development as biosensors? This review examines the identified TFs in light of these questions.

NapA, AtfA, MsnA (ortholog of yeast Msn2/4), and SrrA (ortholog of yeast Skn7) are TFs involved in the oxidative stress response. Upon H_2_O_2_ stimulation, NapA is oxidized by the peroxiredoxin PrxA in the cytoplasm, leading to its translocation into the nucleus to initiate transcription. NapA can be inactivated by thioredoxin TrxA reduction in both the cytoplasm and nucleus [159]. AtfA, a bZIP-type TF, interacts with SakA, the terminal kinase of the high-osmolarity glycerol (HOG) mitogen-activated protein kinase (MAPK) pathway, thereby serving as a key regulator in response to osmotic and oxidative stress [160]. MsnA, involved in various stress responses including oxidative stress, may interact with VelB; however, its upstream signaling components remain unclear. It contains multiple potential phosphorylation and ubiquitination sites, and its DNA-binding motif has recently been resolved [161]. SrrA, a Skn7-type response regulator containing an HSF-like DNA-binding domain and a receiver domain, is essential for H_2_O_2_ tolerance [162]. Nevertheless, information regarding its upstream regulators, downstream DNA-binding motifs, and target genes remains limited. SrbA, a basic helix-loop-helix (bHLH) TF belonging to the sterol regulatory element-binding protein (SREBP) family, has a well-defined DNA-binding motif [163]. The ΔsrbA mutant exhibits impaired growth under hypoxic conditions. Under low oxygen, full-length SrbA located in the endoplasmic reticulum undergoes proteolytic cleavage in a multi-factor dependent process, releasing its N-terminal bHLH domain into the nucleus to function as a transcriptional activator [164].

PacC is a well-studied TF responsive to alkaline pH, and its DNA-binding sequence has been biochemically validated [116]. The upstream regulation of PacC involves the membrane-associated proteins PalI, PalH, and PalF.

HsfA is the master regulator of thermal stress adaptation. Its activity is modulated by phosphorylation, although the specific upstream regulatory kinases remain unidentified. Despite functional conservation across eukaryotes, HsfA homologs exhibit variations in structure, post-translational modifications, and interacting partners [165]. In contrast to the extensive research on heat-responsive TFs, regulators of cold adaptation in fungi are poorly understood. Recently, the transcription factor Scaffold5.t61 was demonstrated to respond to low temperatures and regulate the synthesis of high-quality red pigment via modulation of glutamate metabolism [166].

Deciphering metal ion regulatory networks provides valuable insights for designing biosensors to monitor physiological or environmental metal ions and for developing bioremediation strategies. Ca^2+^ and Zn^2+^ are essential divalent cations involved in diverse biological processes. The primary regulator of calcium homeostasis is CrzA, which has been identified in Aspergillus [167]. In A. fumigatus, ZafA is the zinc-responsive TF responsible for zinc homeostasis and can functionally complement Zap1 in *S. cerevisiae*. Notably, Cd^2+^ can mimic the repressive effect of Zn^2+^ on ZafA. Cu^2+^-responsive TFs are widely reported in filamentous fungi. Mac1 is activated under copper-limiting conditions, whereas trans-activator AceA is induced by high copper concentrations [168,169]. However, specific responsive TFs for other biologically relevant divalent ions such as Mn^2+^, Co^2+^, and Ni^2+^ remain unidentified, although Ca^2+^-CrzA signaling has been implicated in Mn^2+^ sensing.

In fungi, key TFs involved in mechanical sensing and cell wall integrity include Rlm1 and CrzA. Rlm1 is a critical component of the CWI MAPK pathway. Upon mechanical stress or cell wall damage, the CWI pathway is activated, leading to phosphorylation of Rlm1 at specific sites (e.g., Ser427 and Thr435) by Slt2 (Mpk1), which enhances its transcriptional activity [170]. A recent study revealed the DNA-binding sequence of Rlm1 and showed that Rlm1 in *F. graminearum* can be activated by mycotoxins such as deoxynivalenol (DON) [171]. CrzA, a key effector of Ca^2+^–calcineurin signaling, translocates from the cytoplasm to the nucleus upon dephosphorylation in response to calcium stress, promoting the expression of cell wall biosynthesis-related genes. This nuclear translocation can be inhibited by cyclosporine A [172].

**Table 4:** Environmental cue-responsive TF summary.

Inducer	Responsive TF	UAS	Species	Ref.
Oxidative stress				
ROS: NADPH	NapA	(T/TT)ACTAA/TKASTAA	Filamentous fungal species	[173]
	AtfA	DRTGTTGCAA	*A. flavus*	[174]
	MsnA	GCTGAGTCAGC	*A. nidulans*	[161]
Low oxygen	SrbA	(A/G)TCA(T/C/G)(C/G)CCAC(T/C)	*Aspergillus* spp.	[163]
pH				
Alkaline pH	PacC	GCCARG	*A. nidulans*	[116]
Temperature				
Thermal stress	Hsf1	TTCnnGAAnnTTC	*C. albicans*, *Aspergillus* spp.	[175]
Cold	Scaffold5.t61	N/A	*Geomyces *sp.* WNF-15A*	[166]
Metal ion				
Ca^2+^	CrzA	GDGGCKBNB; A[GT][CG]CA[AC][AG]; GGAGGC(G/A)C(T/A)G	*T. reesei*, *A. fumigatus*, *C. albicans*	[176,177,178]
Zn^2+^	ZafA	CAAGGT	*A. fumigatus*	[179]
Cu^2+^	AceA	H(T)HNNGCTGD	*P. chrysosporium*	[180]
Mechanical Forces				
Ca^2+^	Crz1	G[T/G]GGC[T/A]G[T/G]G	*Aspergillus* spp.	[167]
	Rlm1	TGATGCTGTTGATGT, TGCTATTTTTGG	*F. graminearum*	[171]
Water Availability				
Hog1 signaling	AtfA	DRTGTTGCAA	*A. flavus*	[174]

## 5. Biosensors and Synthetic Tools Developed from Filamentous Fungi

**Figure 5:**
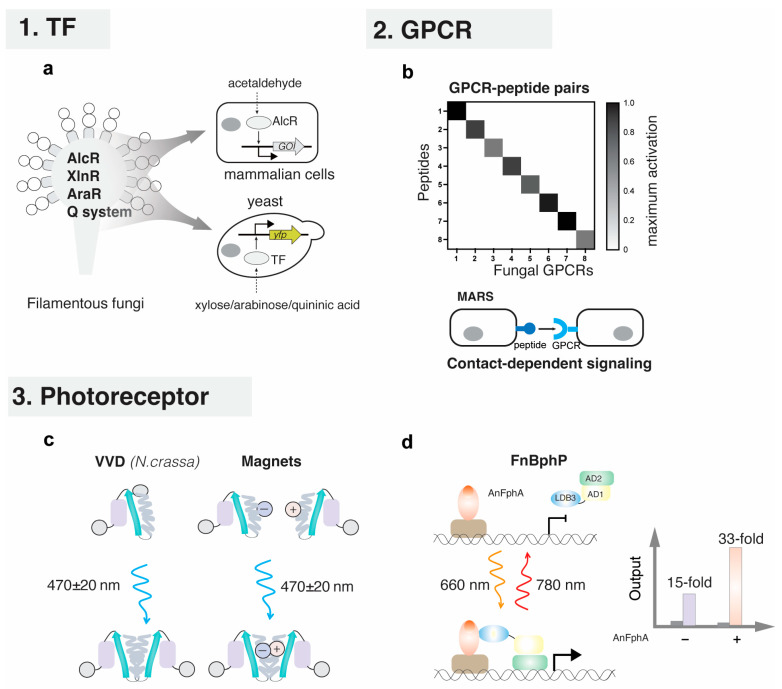
Examples of filamentous fungi-derived genetically encoded biosensors applied in synthetic biology. (**a**) XlnR-, AraR-, and Q system-based biosensors were developed in yeast [20,82] and an AlcR-based biosensor was developed in mammalian cells [75]. (**b**) Fungal orthogonal peptide–GPCR pairs were functional in yeast and applied for the construction of Mating peptide-Anchored Response System (MARS) [21]. (**c**) VVD from *N. crassa* and VVD derivate, Magnets, were engineered as light switches to control protein homodimerization and heterodimerization, respectively [193]. (**d**) The N-terminal extension of FphA from *A. nidulans* was used to improve the sensitivity of the chimeric photosensory proteins [22].

A set of biosensors and synthetic tools has been developed from filamentous fungi. Some examples have been illustrated in Figure 5. These successful cases of cross-species transfer from filamentous fungi have all relied on small-molecule-responsive TFs, pheromone-responsive GPCRs and photoreceptors.

### 5.1. TF-Based Biosensors

Several small-molecule-responsive TFs from filamentous fungi have been demonstrated to be functional in mammalian cells or yeast. In 2004, the ethanol-inducible AlcR-P_alcA_ system from *A. nidulans* was successfully transferred into mammalian cells and was demonstrated not to cross-talk with established antibiotics-induced systems [75]. Furthermore, the Q system from *N. crassa* was introduced in yeast to develop a pair of orthogonal tools, OptoQ-INVRT and OptoQ-AMP, which enable simultaneous optogenetic signal amplification and inversion [82]. Recently, the XlnR-based xylose induction system from *A. nidulans* has been shown to be functional in *S. cerevisiae*, exhibiting superior performance compared to bacterial XylR-based biosensors in terms of inducibility, dynamic range, induction kinetics and impact on host growth [20]. In some cases, although the binding motifs of certain natural responsive transcription factors have never been identified, synthetic TFs can serve as a solution. Synthetic TFs are constructed through modular assembly of DBDs, ligand-binding domains (LBDs) and ADs. For example, since the DNA-binding motif of AraR from *A. niger* was unknown, fusing AraR to the well-characterized LexA DBD enabled the output promoter to be dose-responsive to arabinose [20].

As the mechanisms for sensing and transducing most small molecules have not been fully understood yet, the cross-species transfer of induction systems from eukaryotic cells has long been considered challenging. The functionality of these two-component systems in yeast or mammalian cells suggests that some fungal Zn(II)2Cys6 activators are direct sensors, aligning with a previous study [33], which would greatly facilitate their cross-species transfer.

### 5.2. GPCR-Based Biosensors

The endogenous α-factor pheromone GPCR signaling pathway in *S. cerevisiae* is the most extensively studied one, with its key components’ dose–response relationships having been explored through computational and experimental reconstruction [181]. The yeast α-factor GPCR pathway has become a rational model for heterologous GPCR research. For instance, heterologous expression of orthogonal fungal pathogen GPCRs in yeast enables rapid and sensitive detection of fungal pathogens [182,183]. Furthermore, 32 of 45 GPCRs from Ascomycota have been demonstrated to be functional in yeast, with 17 GPCR–peptide pairs displaying high orthogonality. Using eight orthogonal GPCR–peptide pairs, basic two-cell communication links, complex multicellular communication network topologies and three-member interdependent communities were constructed [184]. Additionally, in a recent study, a yeast toolkit named MARS (Anchored Peptide Response System) was developed based on GPCR-peptide pairs from Ascomycota fungi, including *Beauveria bassiana*, *Trichoderma reesei* and *Saitoella japonica* [21]. These studies show that GPCRs derived from filamentous fungi can be functionally implemented in yeast and applied to pathogenic fungal detection, as well as in complex synthetic topology construction.

### 5.3. Photoreceptor-Based Optogenetic Tools

Optogenetic tools have attracted sustained research interest in neuroscience, immunology and gene expression control due to their operational reversibility and tissue penetrability. Photosensitive proteins derived from filamentous fungi have been engineered to develop synthetic optogenetic tools in mammalian cells and yeast. For instance, in 2012, Yi Yang et al. first constructed the LightOn system by fusing the LOV domain of the *N. crassa* Vivid protein with the Gal4 DBD and the p65 AD. The blue-light-responsive functionality of this system was validated in multiple mammalian cell lines and in mouse livers [185]. The LOV domain was further optimized through mutagenesis to develop a high-performance mutant termed LVADO, providing yeast cells with a powerful and convenient optogenetic tool [186]. In 2015, Moritoshi Sato et al. developed the Magnets system based on Vivid (VVD). By mutating the N-cap domain, they constructed a pair of variants carrying opposite charges, thereby selectively promoting heterodimerization while suppressing homodimerization to achieve ultrafast OFF kinetics [187] (Figure 5c). Furthermore, in 2018, Luis F. Larrondo et al. developed the FUN-LOV optogenetic switch by exploiting the LOV domain interactions between two blue-light photoreceptors from *N. crassa*, WC-1 and VVD [188]. This system exhibits a 1300-fold dynamic range and enables spatiotemporally precise control. Moreover, it was employed to control the expression of the MFα1 gene, thereby achieving optogenetic control of intercellular communication [189].

Beyond *N. crassa*, BcWCL1 from *B. cinerea* requires blue-light stimulation for interaction with BcWCL2 upon deletion of its PAS domain, suggesting their potential as novel optogenetic switch elements [190]. Additionally, another photoreceptor from this species, BcLOV4, was found to translocate from the cytoplasm to the plasma membrane within approximately one second upon blue-light (455 nm) illumination, and this process is fully reversible [191]. Interestingly, in 2023, this protein was further reported to not only translocate to the membrane but also to form clusters on the membrane upon blue-light stimulation. BcLOV4 thus represents the second photoreceptor reported, following Arabidopsis Cry2, whose light-induced clustering on the membrane could be exploited for extensive use [192]. Recently, Haifeng Ye et al. engineered a chimeric photoreceptor, FnBphP, by fusing the N-terminal extension of the *A. nidulans* phytochrome FphA with the photosensory core module of *Deinococcus radiodurans* BphP, thereby developing a sensitive red-light-inducible dimerization system. Compared to DrBphP, FnBphP containing the FphA-NTE significantly enhanced activation levels at 660 nm [22].

Taken together, a growing number of photoreceptors derived from filamentous fungi are being characterized and applied in optogenetic tool development, highlighting their importance in optogenetics.

## 6. Concluding Remark and Future Perspectives

As critical components for signal sensing, GPCRs, TFs and photoreceptors represent fundamental genetic parts in biosensor development. Due to the inherent limitations of prokaryotic components, the development of eukaryotic parts derived from filamentous fungi that are orthogonal to yeast should receive greater attention. Compared to yeast, genetic components involved in unique phenotypes of filamentous fungi—such as extensive nutrient degradation, secondary metabolite synthesis and light perception—may provide diversity to the yeast toolkit. Indeed, signal transduction pathways for both small molecules and environmental factors are considerably more complex in eukaryotes than in bacteria; however, the growing number of successful cases for cross-species transfer of one- or two-component elements from filamentous fungi suggests that mining biosensors from these organisms is indeed feasible and more accessible than previously recognized. Biosensors developed from these components are expected to address the limitations commonly faced by bacterial biosensors, including low-level induction, host cell toxicity, and limited space for performance optimization.

Despite the promising prospects of developing biosensors from filamentous fungi, several challenges limit their development. First, the vast majority of filamentous fungal biosensing elements remain uncharacterized, representing a critical bottleneck for scalable biosensor development from these organisms. Therefore, it is essential to systematically dissect ligand–GPCR and ligand–TF interactions, as well as elucidate the functional domain of photoreceptors in filamentous fungi. For characterization of ligand–GPCR pairs, established high-throughput platforms are available for reference, such as DCyFIR technology [194] and yWS677 and yWS1922 model yeast strains [181]. Regarding the dissection of ligand–TF pairs, recent mapping of the TF–ligand interaction landscape in *E. coli*, employing a methodology interweaving experimental validation and Artificial Intelligence-assisted computational prediction, may serve as a reference [195]. Second, developing specific environmental-factor-responsive biosensors could prove challenging due to complex transduction pathways and crosstalk. Nevertheless, designing these biosensors is important for at least two reasons: (1) precision metabolic engineering requires not only monitoring but also automatic regulation driven by intracellular physiological signals; (2) the predominant environment-factor biosensors are developed based on optical transduction mechanisms, which suffer from challenges in field applications [196]. Therefore, it is essential to enhance research on signaling mechanisms of environmental cues to circumvent current bottlenecks.

The application scenarios for biosensors are extremely broad, spanning the biomedical sciences and material sciences. Biosensors can be integrated to facilitate drug discovery and screening, intelligent drug delivery, therapeutic drug monitoring, point-of-care drug/pathogen diagnostics, and living material fabrication. For example, filamentous fungi are well known for producing key pharmaceuticals, including penicillin, griseofulvin, lovastatin, cyclosporine, and ergometrine. Monitoring their pharmacokinetics is necessary due to potential issues with dosage and toxic side effects. In the long term, drug-responsive genetically encoded biosensors developed from filamentous fungi could facilitate in vivo monitoring and personalized therapy.

## Data Availability

No new data were created or analyzed in this study. Data sharing is not applicable to this article.
